# A Bayesian Mixed Regression Based Prediction of Quantitative Traits from Molecular Marker and Gene Expression Data

**DOI:** 10.1371/journal.pone.0026959

**Published:** 2011-11-07

**Authors:** Madhuchhanda Bhattacharjee, Mikko J. Sillanpää

**Affiliations:** 1 Department of Statistics, University of Pune, Pune, Maharashtra, India; 2 Department of Mathematics and Statistics, University of Helsinki, Helsinki, Finland; 3 Department of Agricultural Sciences, University of Helsinki, Helsinki, Finland; 4 Department of Mathematical Sciences, University of Oulu, Oulu, Finland; 5 Department of Biology, University of Oulu, Oulu, Finland; University of Turin, Italy

## Abstract

Both molecular marker and gene expression data were considered alone as well as jointly to serve as additive predictors for two pathogen-activity-phenotypes in real recombinant inbred lines of soybean. For unobserved phenotype prediction, we used a Bayesian hierarchical regression modeling, where the number of possible predictors in the model was controlled by different selection strategies tested. Our initial findings were submitted for DREAM5 (the 5th Dialogue on Reverse Engineering Assessment and Methods challenge) and were judged to be the best in sub-challenge B3 wherein both functional genomic and genetic data were used to predict the phenotypes. In this work we further improve upon this previous work by considering various predictor selection strategies and cross-validation was used to measure accuracy of in-data and out-data predictions. The results from various model choices indicate that for this data use of both data types (namely functional genomic and genetic) simultaneously improves out-data prediction accuracy. Adequate goodness-of-fit can be easily achieved with more complex models for both phenotypes, since the number of potential predictors is large and the sample size is not small. We also further studied gene-set enrichment (for continuous phenotype) in the biological process in question and chromosomal enrichment of the gene set. The methodological contribution of this paper is in exploration of variable selection techniques to alleviate the problem of over-fitting. Different strategies based on the nature of covariates were explored and all methods were implemented under the Bayesian hierarchical modeling framework with indicator-based covariate selection. All the models based in careful variable selection procedure were found to produce significant results based on permutation test.

## Introduction

The development of efficient statistical methods which can provide accurate prediction of the unobserved phenotype based on genomic profile of an individual is the target in many research fields including human, animal and plant genetics [1]–[3]. Phenotype prediction methods are often based on classification and regression trees [4]. There has been recent interest to apply Bayesian variable selection [5] and frequentist regularization methods [6] to perform parameter estimation and variable selection simultaneously in phenotype-genotype and phenotype-expression association analyses. These methods also performed well in selecting important subset of trait-associated loci to estimate genomic breeding values in animals and plants [7]–[10]. Lee *et al.* (2008) [2] considered that methods for predictions of unobserved phenotypes and genomic breeding values have same goal and can be successfully substituted for one another. Typically such prediction methods consider a single type of genomic data (molecular marker, gene expression or protein expression) for prediction at a time even if prediction accuracy may be improved by considering multiple data types simultaneously [1].

Rapid advancements in laboratory techniques have made a cheap production of a gigantic amount of genomic molecular marker and expression data (that is putative predictors) possible. The simple statistical screening methods to find phenotype-genotype association or phenotype-expression association are still much used in practice because high dimensionality of the genomic data prevents use of more advanced statistical variable selection methods due to their computational demands. This state-of-the-art data combined with use of outdated statistical tools controversy have made a question of applicability of dimension reduction techniques very acute. Thus, several statistical initial screening methods have been developed which can filter large sets of predictors to the smaller sized sets so that more advanced methods can be applied to the selected predictors in the subsequent stage. By reducing size of the predictor set, the variable selection problem becomes less ill-posed as number of predictors is starting to exceed the number of individuals. Also a technique called “preconditioning” which can reduce noise from the variable selection experiments have received substantial attention recently among statisticians (see [11]–[12]). However, in such two-step procedures, the most common reduction tool in practice seems to still be a simple correlation coefficient calculated between the phenotype and the putative candidate (marker locus or gene expression) in question.

In this paper, we have applied Bayesian methods in the context of association mapping to predict unobserved phenotype values of the soy plants with single feature polymorphism (SFP) genotype data, gene expression data and also both of these data types combined. We will loosely refer to the genomic locations like SFPs and genes as “markers”. Prediction is done using Bayesian models which perform simultaneous variable selection and parameter estimation. Our predictions were ranked as the best performing results for sub-challenge B3 of DREAM5 (the 5th Dialogue on Reverse Engineering Assessment and Methods challenge). The further improvements of these models are presented here and their accuracies are assessed in comparison to our original B3 models.

## Materials and Methods

### Materials

The data explored here is based on genetic and functional genomic studies on soybean provided as a part of DREAM5. The data sets along with descriptions are available from http://wiki.c2b2.columbia.edu/dream/index.php/Challenges and in [13] where the mechanism of data generation has been described.

Recombinant Inbred Lines (RILs) were produced by many generations of selfing starting with two distinctly different inbred lines with substantial difference in susceptibility towards a major pathogen. Genotype measurements, in the form of single feature polymorphism (SFP), were available for 941 locations on the genome. Gene expression measurements on 28395 genes were also available. In all, SFP measurements were available on 260 plants and gene expression measurements were available for an overlapping set of 260 plants. There were 230 common individuals for whom both SFP and gene expression measurements were available.

The response variables in this data were two different measurements on pathogen activity, which will be referred to as “phenotype(s)”. Both the phenotypes represent measures of amount of pathogen in the infected tissue sample and relate to severity of infection. The first phenotype (denoted as phenotype-1) is measured as ‘percent present’ and the second (denoted as phenotype-2) is captured as ‘scale factor’. Interestingly both the phenotypes were measured on continuous scale and their distributions were approximately normal. Because of this, we use parametric Pearson correlation coefficient instead of Spearman correlation for accuracy assessment in following if not stated otherwise.

**Figure 1:**
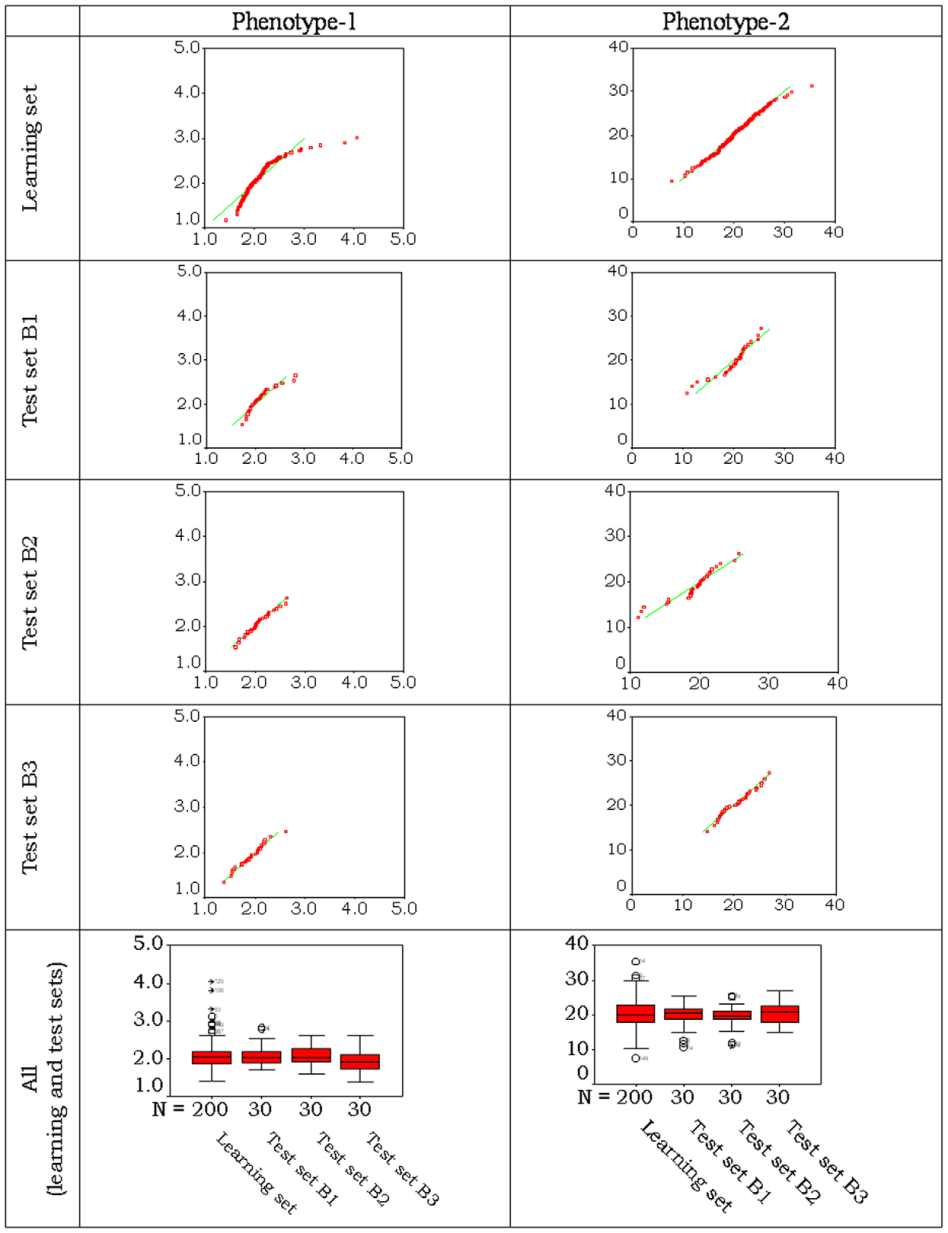
Normal Q–Q plots and Box-plots for the given sets of data. For Q–Q plot the observed values are in X-axes and expected normal values are in Y-axes.

The original data sets came with suggested learning and test sets (given split-sample data) for carrying out various predictive exercises. The original requirement of the challenge was to analyse the split-sample data provided and the best results were obtained by us and these were 0.28 (0.19) and 0.24 (0.18) for the two phenotypes according to Spearman (Pearson) correlation. However initial exploratory analyses of the corresponding subsets of phenotype data suggested that these sets unfortunately may differ from each other in their underlying statistical distributions, although the sets were created by random sub-sampling (Figure 1). Thus, prediction accuracies of the methods in following are assessed by using a generalized strategy.

**Figure 2:**
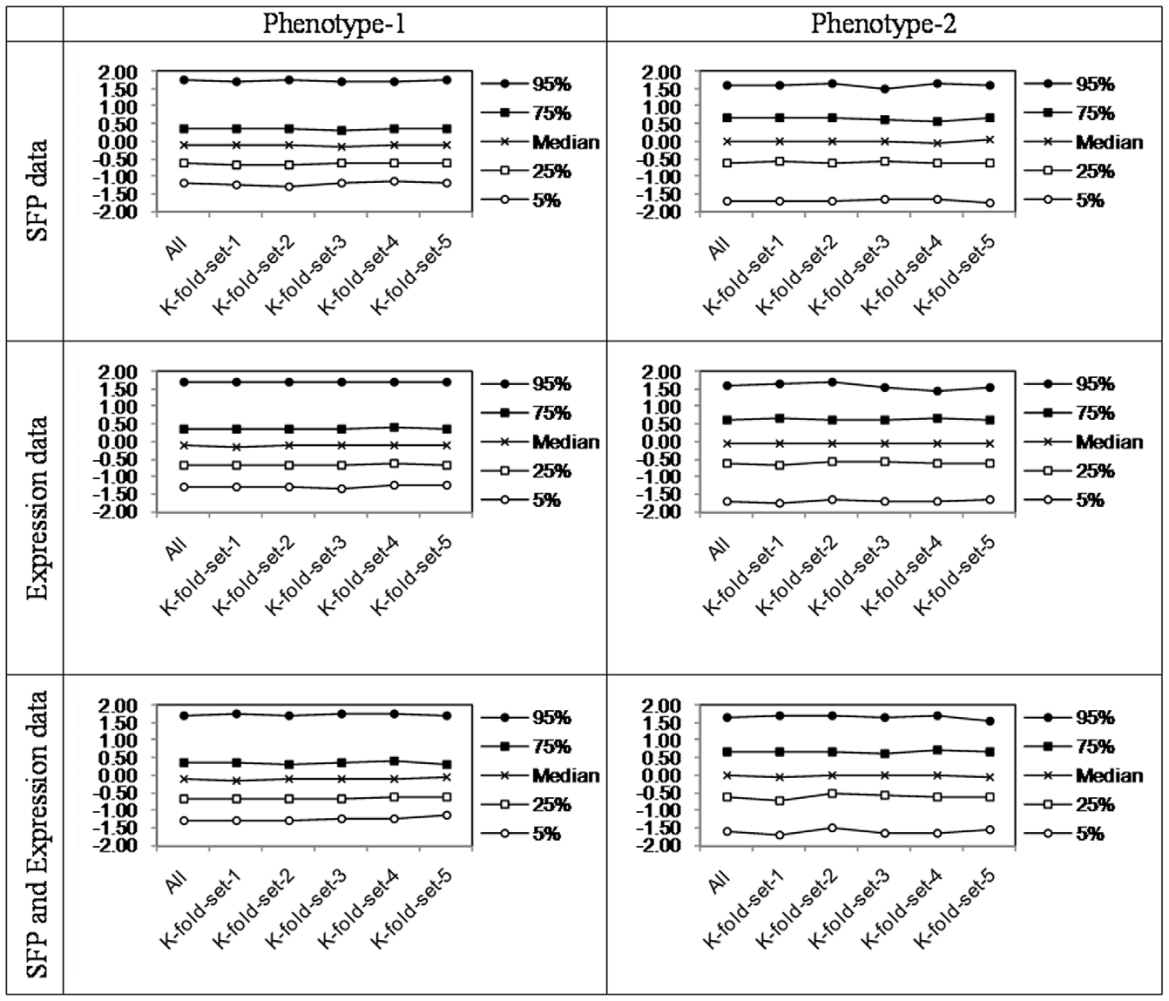
Percentile distributions of the original data and 5-folds created for k( = 5)-fold cross validation.

The prediction assessment strategy as suggested originally is known as split-sample or hold-out method. A generalization of this technique, which is also known to be more powerful, is *k*-fold cross-validation method [14]-[15]. In this process the entire data set is split into *k-*(near) equal subsets and prediction algorithm is trained on the data separately *k*-times each time leaving one subset out. Prediction is then assessed by measuring the predicted outcome/response of the unseen part of the data not used for training. Ideally training set should be as large as possible; however this also means that the size of test set will be small. In order to exhaust the entire data covering by small test sets would imply many repetitions of the training and prediction procedure, increasing the computing time linearly. As a compromise between learning set size and computation time we chose *k = 5*. Accordingly learning sets were created randomly, however post checks were made to ensure the all the subsets of phenotypes (the main variables of interest) have similar distribution on all *k*-sets (Figure 2).

### Methods

In following, we will consider three different multilocus regression models depending on the type of the covariate data included into the model: (1) only SFP data, (2) only gene expression values, or (3) both SFP and gene expression data jointly. We also carried out different strategies to select important covariates into the regression models. Summary of these are presented in Table 1, where the columns from left to right presents a rough flow-diagram of the analysis procedure.

**Table 1:** Correlation of prediction for different variable selection processes and model types are presented according to data types (carried out separately for each phenotype).

Cross validation subset	Variable selection	Further data reduction	Model	In-data/goodness of fit		Out-data/out-of-sample prediction	
				Pheno-type-1	Pheno-type-2	Pheno-type-1	Pheno-type-2
SFP-data							
Split-sample	None	Shrinkage^3^	Indicator model	**0.84**	**0.83**	−0.05	0.08
				**(0.74)**	**(0.81)**	(−0.12)	(0.01)
	Best performance in DREAM5			NA	NA	NA	NA
*k* ( = 5) fold cross validation	T-test based^1^	None	Non-indicator model^7^	0.44	0.49	0.33	**0.26**
				(0.34)	(0.47)	(0.25)	**(0.26)**
		Vague prior^4^	Indicator model^8^	0.44	0.59	**0.33**	0.25
				(0.34)	(0.56)	**(0.25)**	(0.25)
Expression-data							
Split-sample	Correlation based^2^	Shrinkage^3^	Indicator model	0.79	0.88	0.22	−0.06
				(0.77)	(0.86)	(0.26)	(0.03)
	Best performance in DREAM5			NA	NA	(0.31)	(0.26)
*k* ( = 5) fold cross validation	Correlation based^2^	Supervised PCA^5^	Non-indicator model	**1.00**	**1.00**	0.36	0.37
				**(0.88)**	**(0.88)**	(0.32)	(0.32)
		Common subset selection^6^	Non-indicator model^7^	0.48	0.71	**0.41**	0.47
				(0.42)	(0.66)	**(0.36)**	(0.38)
		Common subset selection^6^ and vague prior^4^	Indicator model^8^	0.46	0.69	0.39	**0.47**
				(0.40)	(0.62)	(0.33)	**(0.38)**
SFP and Expression-data							
Split-sample	Genes: Correlation based^2^	Shrinkage^3^	Indicator model	**0.91**	**0.94**	0.19	0.18
				**(0.91)**	**(0.92)**	(0.31)	(0.24)
	Best performance in DREAM5			NA	NA	(0.31)	(0.24)
*k* ( = 5) fold cross validation	SFP: T-test based^1^ Genes: Correlation based^2^	Common subset selection^6^	Non-indicator model^7^	0.63	0.87	**0.52**	**0.50**
				(0.57)	(0.84)	**(0.48)**	**(0.45)**
		Common subset selection^6^ and vague prior^4^	Indicator model^8^	0.61	0.77	0.48	0.47
				(0.56)	(0.73)	(0.44)	(0.42)

Pearson-correlation is presented first followed by Spearman correlation within brackets.

1 SFPs are ranked according to their (absolute) *t*-statistics (marginal) and entries are selected from top.

2 Genes are ranked according to their (absolute) correlation between expression and phenotype. The top 10% were selected. Expression information on 260 plants used for this purpose.

3 Shrinkage parameter based (*a-priori* independent) prior distribution for inclusion-indicator variable in model was used with shrinkage of 0.1 for SFPs and 0.01 for gene expression data.

4 Vague/Uniform(0,1) prior distribution for inclusion-indicator variable in model was used for individual SFP/gene.

5 Top components from PCA of the gene expression data involving only those genes selected first based on phenotype/expression correlation.

6 Correlations of expression with phenotype were computed for each gene based on a) all 260 plants in the data b) also for each of the 5 learning sets created by 5-fold cross validation. Genes common in these 6 sets with highest correlation were identified and top subsets used for analysis.

7 The results presented are the best using a top subset for each phenotype separately. The cumulative top sets were created and explored for prediction with up to 50 SFPs and/or 100 gene expression measurements.

8 Predictive results obtained using the same top subset producing best results with a non-indicator model, however since the model is indicator based the effective number of covariates are less than that used in the non-indicator based model.

### Predictive model

The association model in general for individual 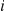 can be written as:
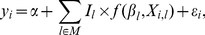
(1)where *M* is the set of all markers (i.e. SFPs and/or gene expressions), *X* = (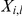) is the matrix of observed data with (*i,l*)^th^ entry corresponding to that of plant *i* and marker *l*. In case of gene expression data, these entries contain values of transcription abundances and in SFPs, they are genotype codes with numbering depending on the parameterization of the model; for example, value zero (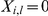) may correspond to the one genotype and the value one (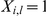) for the other genotype. If a direct constraint on the 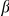 parameters are used then coding for genotypes are used to identify appropriate coefficient and thus is flexible. Whereas if 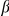 are unconstrained then the entries in X could be binary to implement a constraint on betas. The function *f* is chosen appropriately depending on the nature of explanatory variables in *X*. For expression data and SFP data with genotype codes zero and one, it is simply 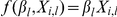. The parameter 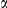 represents the intercept, 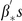 are the coefficients, the latent variables 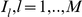 govern inclusions/exclusions of the explanatory variables into the model and 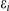 are the independent error terms following normal distribution with zero mean and unknown variance 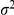.

### Pre-selection of the best ranking predictors

In preliminary study (Table 1) it was found that use of all 941 SFPs/2840 gene expressions in the model provides excellent in-data predictions (i.e., goodness-of-fit) but not satisfactory out-data predictions. Thus this indicated that a possible problem of over-fitting has occurred. Therefore we focus on various pre-selection methods described below. Inclusion of the selected covariates in to the model is either directly governed by a random variable taking 0–1 values (referred as indicator models) or otherwise inclusion or exclusion of each predictor was prefixed (referred as non-indicator model). Note that non-indicator models can also be expressed as a special case of indicator model using degenerate random variables for indicators.

Thus all the proposed models can be expressed as Bayesian regression model with spike-n-slab method for variable selection similar to those proposed for association models [16]–[18] using subset of the markers to predict phenotype value (*y_i_*) of an individual plant 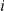. We assume *a-priori* that there is only a small subset of important markers that are useful to predict the phenotype. In these predictive models, subset selection of important marker effects to the predictive model (after pre-selection) is based on use of random indicator variables to be estimated (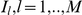), all of which can be either equal to one (inclusion) or zero (exclusion) depending on the importance of particular SFP. Here, 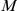 is a number of markers.

#### Priors for α, β, I and 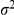


Prior specification is intrinsically subjective and specifying prior that will satisfy everyone and/or every aspect might be un-achievable. We adopt the method where priors reflect our intuitive knowledge but are also useful in avoiding some potential pitfalls and helping to reduce the computational burden. Typically, we assume Gaussian errors with unknown variance under inverse-Gamma distributed prior. We assume a standard Normal prior distribution for the intercept parameter *α*. The coefficient parameters (marker effects) are also assumed to be Gaussian with marker and marker type specific distributional choice for the variance parameters. Typically in indicator-based variable selection models the prior probability for each SFP to be involved in the model is 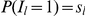, where 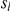 can be: a) a given constant or fractional value (where use of small value means strong shrinkage and sparse model representation) depending on the type of a marker, b) given extreme values of 0 or 1 and c) assumed to be Uniformly distributed random variable between zero and one. Choice (b) above enables non-indicator models to become special case of indicator-based models.

The models were implemented in WinBUGS [19] software which is specialized software to carry out Markov chain Monte Carlo simulation from posterior distribution of complex models.

### Variable selection and data- reduction efforts

It is known that including only the most associated subset of trait loci (say, 5–15 markers) from the genome-wide association studies to the predictive model suffers from low predictive power [20]. Also for small and noisy data sets, common phenomena known as over-fitting (i.e., a model shows good fit for the data at hand but provides poor predictions for unseen data) may easily occur [21] when number of covariates is rather large even if model is simple (e.g. as basic a model as regression). Both these problems can be reduced by appropriate choice of covariates/explanatory variables. For association studies, applications of state-of-the-art statistical variable selection methods have been carried out wherein the models include all markers simultaneously [22]–[23]. It is also known that in high-dimensional problems with small sample size, use of pre-selection methods (e.g., variable ranking methods; [24]) to carefully select subset of predictors as inputs for more advanced methods improves predictions [25].

The kind of data that we have used here is becoming more commonly available and it has both the above aspects, that is studying association aspect is of importance as well as putative covariates from high throughput techniques are available where actual sample size is comparatively not large. Thus exploring appropriate variable selection becomes essential for useful further application of models thus developed. The following alternative strategies were considered.

### SFP data-shrinkage

The size of the original marker set was not large, covering 941 SFP measurements for each plant, and thus drastic initial steps to reduce size may not be necessary. In our initial attempt, a shrinkage parameter value of 1/10 was used as prior probability of inclusion into the regression model for each SFP individually (see model 1 above). This corresponds to *a-priori* assumption of approximately 100 SFPs to be effective in the model.

### SFP data-*t* statistics

Alternately SFPs are ranked according to their (absolute) value of the *t*-statistic which was used to measure the marginal relevance of an SFP to predict a phenotype and this is repeated for each phenotype separately. The two phenotypes produced rather different rankings of the SFPs according to this criterion. The cumulatively chosen top subsets of SFPs were then used in the predictive model for each individual phenotype separately.

### SFP data-Indicators with vague-prior

The *t*-statistic described above is only measuring marginal predictive performance and therefore a subset of SFPs selected based on their marginal significance may result including some redundant predictors into the model. Thus in addition to the *t*-statistics a further reduction or control on inclusion can be achieved by assigning indicator variables to control inclusion of markers in the model. The indicator variables were assumed to have inclusion probabilities distributed as *Uniform(0,1)* distribution. Thus if a subset of size *k* top SFPs are included in the model then prior inclusion probability further assumes *a-priori* that only *k/2* markers are effective in the model.

### Expression data – phenotype/expression-correlation

The genes are ranked according to their (absolute) correlation between transcript abundance and phenotype measurements over the plants. Information on all 260 plants, with expression data, were used for this purpose and top 10% genes were selected as a primary set of genes for all expression data related analyses. Once again the lists were specific for each phenotype.

### Expression data - shrinkage

Similar to the SFP data shrinkage parameter based on (*a-priori* independent) degenerate prior distribution for inclusion of a gene in the model was used with prior shrinkage probability of 0.01 for gene expression data.

### Expression data-subset selection

The *k*-fold cross validation method creates *k* learning sets and phenotype/expression correlation for each of these subsets of individuals (in a particular learning set) was derived. A surprising amount of variability was noticed amongst the top genes thus listed. Only 237 genes for phenotype-1 and 180 genes for phenotype-2 were common in all 5 subsets amongst the top 5% genes. Then top genes common in all 5-lists were cumulatively taken to form subsets (for each individual phenotype separately). Alternatively, bootstrap re-sampling technique may be used to estimate the variability in phenotype/expression correlation of genes. In this method, the selection procedure would be independent of choice of specific *k*-fold stratification of the data and genes found to have high variation among the (correlation-wise) top genes may be avoided for further analysis. This strategy is somewhat related to so called “stability selection” of [26] which considers variable selection problem to decide which subset of variables to choose while our interest here is in out-of-sample prediction. While the above strategy is fine from variable selection point of view, it suffers from “use-of-data-twice” type of treatment from prediction point of view. This is because the test set data is already used once to determine variability of the candidates in the learning stage. For possible solution, see the Discussion section.

### Expression data-Indicators with vague-prior

Similar to marginal testing of SFPs, subset selection based on the highest phenotype/expression correlation will also result in some redundancy among selected predictors in the model. Similar to that applied to the SFPs, further stringency on inclusion in the model is attempted for gene expressions by introducing suitable indicator variables with vague prior probabilities.

### Expression data – supervised PCA

Principal Component Analysis (PCA) was carried out for the expression measurements of the top 10% genes first selected based on the highest marginal phenotype/expression correlation. The top principal components explaining majority of the phenotypic variation are then used as predictors in the model. This method is known as supervised PCA [27].

**Figure 3:**
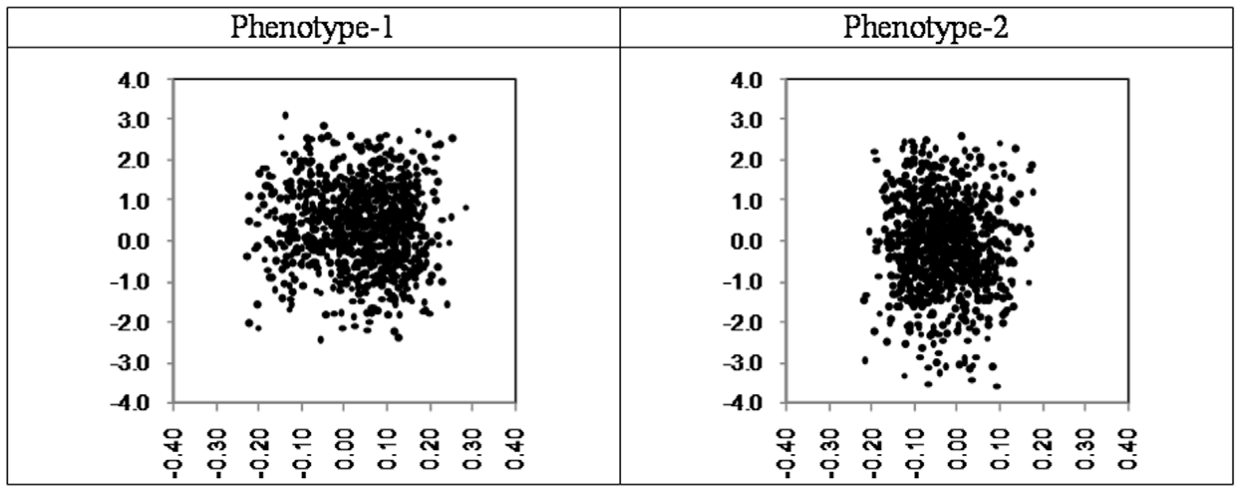
Scatter plots of SFP-specific t-statistics and phenotype/expression correlations of the probes common between the SFP data and gene expression data.

It was noted that most of the genes pertaining to the 941 SFPs on which genetic data was collected were also present in the set of genes for which expression measurements were collected. As an essential part of variable reduction effort it was investigated if the information conveyed by the two types of data has redundancy so that only unique information from both can be used. However the marginal signals as captured by the *t*-statistics of the SFPs and phenotype/expression correlations of the genes have no evident relationship/connection with each other in two phenotypes (see Figure 3). In the scatter plots of Figure 3, pairing of each SFP and gene expression was done based on the common probes. Thus, both types of data on these common genes were considered to carry complementary information on further analyses.

### Sampling variability related issues

It is known that because of high degree of variability in expression of genes across samples, it is often difficult to capture a truly representative sample through handful of subjects. The current data make no exception in this.

To assess the effect of variability of gene expression on the predictive ability of the respective gene, we carried out a bootstrap re-sampling exercise. Predictive ability of a gene was quantified by a (Pearson) correlation between transcript abundance and phenotype over the re-sampled plant data sets of each gene. This effort indicated the likely independence between the mean and the standard deviation of such correlations. That is a gene can have on an average high correlation with phenotype but could have any possible variation (across re-sampled data sets) and similarly the most stable genes (across re-sampled data sets) can have any degree of predictive ability (See Figure S1 and Text S1 in Supporting Information). In such data sets, good performance in the sense of having high correlation and high stability at the same time would be difficult to achieve without utilizing test set information already in the learning stage as was done above in the *Expression data-subset selection*.

## Results

### Prediction based on SFP data only

**Figure 4:**
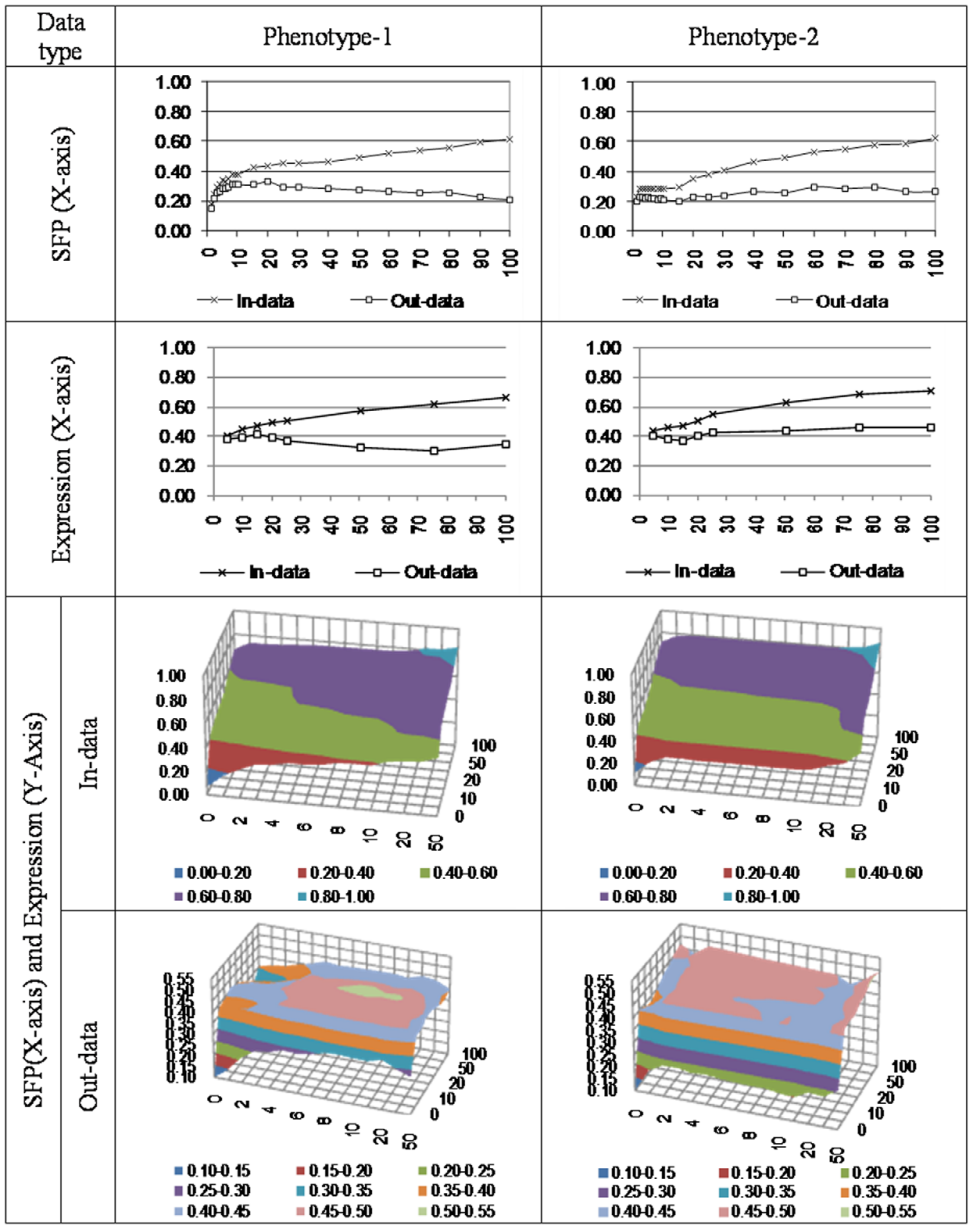
Correlations calculated between observed and predicted phenotypes with varying numbers of covariates in the model.

**Figure 5:**
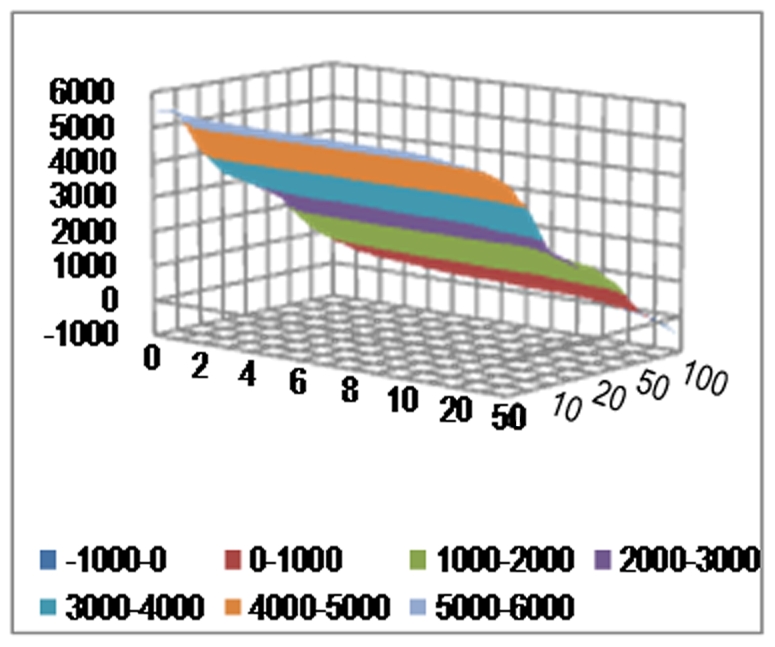
Deviance (in vertical axis) with varying number of SFPs (in X-axis) and expression data (in Y-axis) into the model.

Correlations between observed and predicted phenotype values in Figure 4 indicate the following: In-data prediction for both phenotypes improves with increase in number of SFPs in the model. Out-data prediction for phenotype-2 improves (in general) with larger number of SFPs in the model. Out-data prediction for phenotype-1 however improves only up to a small number of SFPs (around 15–20) in the model. As expected, deviance always seems to improve with more SFPs in model which basically reflects the gain in goodness-of-fit or quality of in-data prediction (Figure 5).

### Prediction based on only expression data

Correlations between observed and predicted phenotype values indicate that in-data prediction for both phenotypes improves with increase in number of genes in the model (Figure 4). Out-data prediction for phenotype-2 improves (in general) with larger number of genes in the model. Out-data prediction for phenotype-1 however behaves non-monotonically with increase in number of genes. As before deviance improves with more complex model (Figure 5).

### Prediction based on joint SFP and expression data

As before the picture based on correlations between observed and predicted phenotype lead us to conclusions similar to above: including more predictors in the model improves prediction accuracy (Figure 4). Phenotype-2 out-of-sample prediction does get better with increased complexity/size of model, whereas limited number of markers are preferred for phenotype-1. Goodness-of-fit as measured in-data prediction or deviance improves with increase in number of either SFP or genes with expression data (Figure 5).

From different data types analyzed here, we can roughly say that, use of both data types simultaneously improves out-data prediction accuracy compared to the accuracy obtained using only a single data type at a time (Table 1). This is in line with suggestion of [1], but differs from what we have earlier seen in association study context [17] or with the view that impact of genetic polymorphisms on phenotypes operate indirectly via the gene expressions (intermediate phenotypes) [28]–[29]. In terms of Pearson correlation coefficient, prediction accuracy for phenotype-1 improved from 0.41 to 0.52 in one of our models and from 0.39 to 0.48 in another model (Table 1). For phenotype-2, we do not see similar advantage for out-sample prediction accuracy as for phenotype-1.

### Benefits of Variable Selection

The models attempting to use entire or most part of the data without adequate subset selection produces near perfect within-data fit, but they could easily perform poorly in prediction of unseen data (Table 1). Thus, a careful subset selection of predictors would be essential in building a good predictive model which is also visible in our results.

After applying *t*-test/correlation based pre-selection of the markers we further compared performances of the indicator models (i.e. with random variable prior probability) and non-indicator models (i.e. degenerate distribution with extreme values for prior probability) with different choices of parameters. It appeared that if the effective numbers of covariates are kept same in indicator and non-indicator model the indicator model produces better in-data prediction and comparable out-data prediction. This is not surprising since to make the effective number of covariates comparable in the two models the potential set of SFP and/genes for indicator model will be larger allowing the model to explore more complex models yielding better in-data prediction. To remove this added advantage of the indicator models and make the comparison more stringent we applied indicator models on the same subsets of SFPs and/genes that produced the best results (among the ones explored here) with the non-indicator model. It should be noted that thus the indicator models effectively uses approximately only half the covariates due to the added prior on indicator probability. Surprisingly the indicator models produced equally good or comparable results for out-data prediction (Table 1).

It is expected that since the indicator-based model is able to use and explore larger set of prospective predictors compared to (fixed/pre-specified) non-indicator model it would be able to provide insight in choice of preferable sets of covariates. Unfortunately from this aspect the results on this particular data were not helpful.

**Figure 6:**
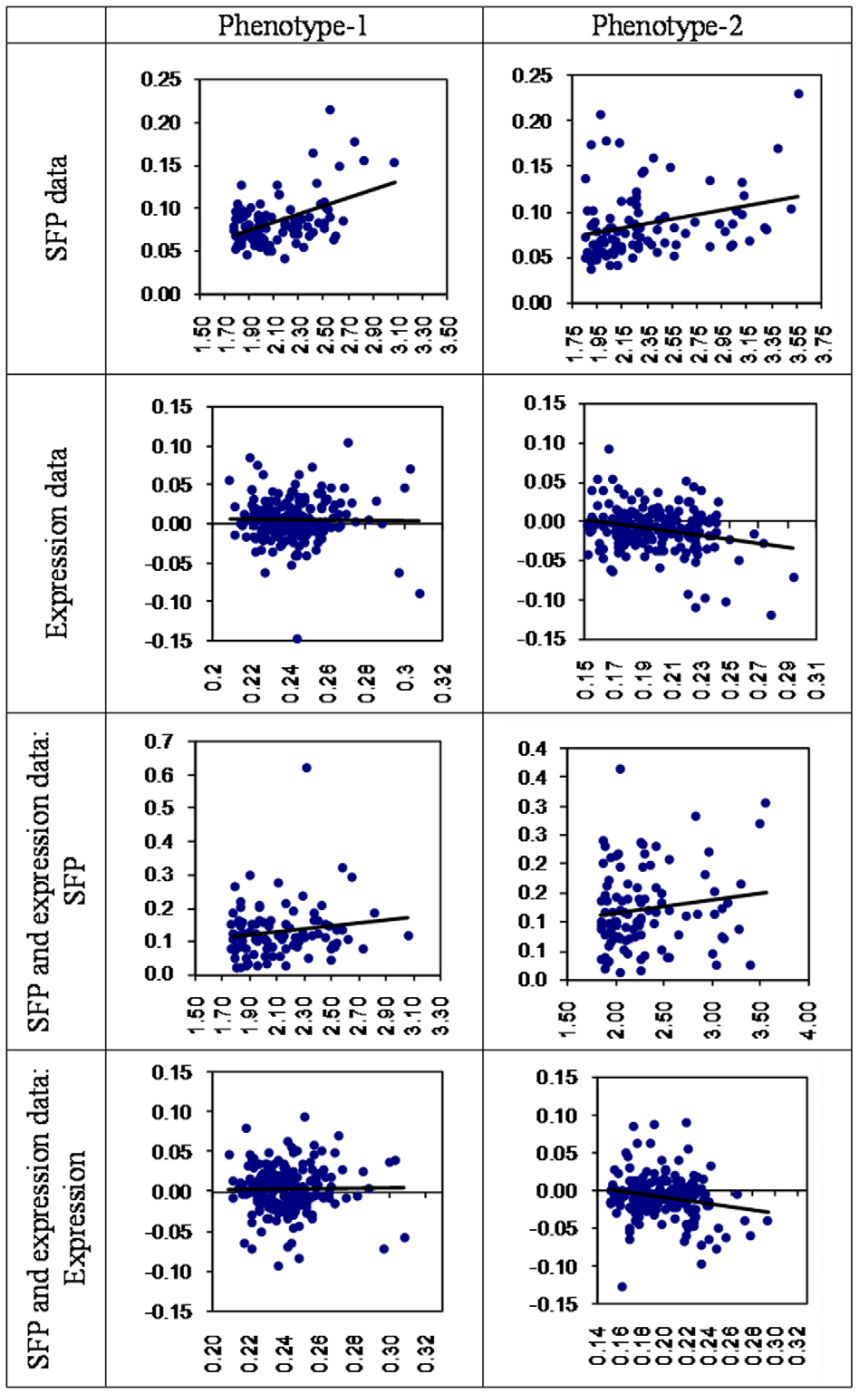
Comparison of variable selection measures. In axis (absolute) t-statistic/correlation (i.e. marginal estimates) are presented and in Y-axis estimated weighted genetic variation (for SFPs) or weighted coefficients (for genes) from joint distribution based on vague priors (i.e. joint estimates) are presented.

However comparison of variable selection measures as given by *t*-test/(absolute) correlation (i.e., marginal estimates) and posterior estimates of relevance from joint distribution based on vague priors (i.e. joint estimates) based on indicator models were carried out. The relevance measures used here are weighted genetic variation for SFPs [16] and weighted coefficient for gene with expression measurements. These quantities are simply calculated as indicator times the coefficient (or the absolute difference between two coefficients). As we know the joint and marginal behaviors does not necessarily need to have any relationship. However, there are linear relationships with significant coefficients in most cases, in particular for phenotype-1 (Figure 6), noted exception being for phenotype models involving gene expression data. Thus although a direct application of indicator model was not useful in providing information on the overall number of variables that would suffice to predict the phenotypes well, still the estimated inclusion probabilities of individual predictors show partial concordance with the marginally selected sets of predictors. This could be indicative that large number of interaction effects may not be present in the underlying genetic architecture of the trait. A smaller but significant set of interactions might of course be very well useful.

### Gene-set enrichment analysis

Most of the published works on gene-set enrichment analysis are based on situations where phenotypes have distinct categories of outcome, like cancer types, or treatment/control etc [30]. However for the current problem the phenotype of interest has continuous outcome, thus making it difficult to obtain single measurements representing enrichment of those gene sets that are meaningful biologically and also contributes critically in “prediction of the phenotype”.

Thus, we studied chromosomal level enrichment of genes and enrichment of the gene sets involved in different biological processes [31]. This was done by collecting Gene Ontology (GO) and chromosomal annotations from public databases for the genes on which we have expressions or SFP measurements available in the current data set. The different nature of the explanatory covariates used in the model, *viz*. SFP and expression data, made it further difficult to provide a consolidated enrichment picture of the underlying processes.

Therefore we summarized the enrichment of these genes from two complimentary contributions by them. Based on the genetic data the summary measure reflects criticality of the average genetic variation in a particular gene-sets in prediction of phenotype. This is measured by the average weighted genetic variation of the SFPs involved in the process. The expression data on the other hand enables us capture the functional variability of the genes involved in a process. The coefficient of a particular gene in the predictive model captures individual contribution of that gene in the prediction. However these when averaged over several genes involved in a pathway might present altogether a different picture. This is measured by the weighted coefficients for the genes involved in the process and was calculated jointly for the gene set.

Firstly note that both these measures include probability of enrichment as well as magnitude of enrichment. Secondly the contribution of any gene/marker could very well be affected by presence or absence of other genes and/or markers in the model. As a result, as we will see in subsequent exploration, results and interpretations may very well have to be context specific.

**Figure 7:**
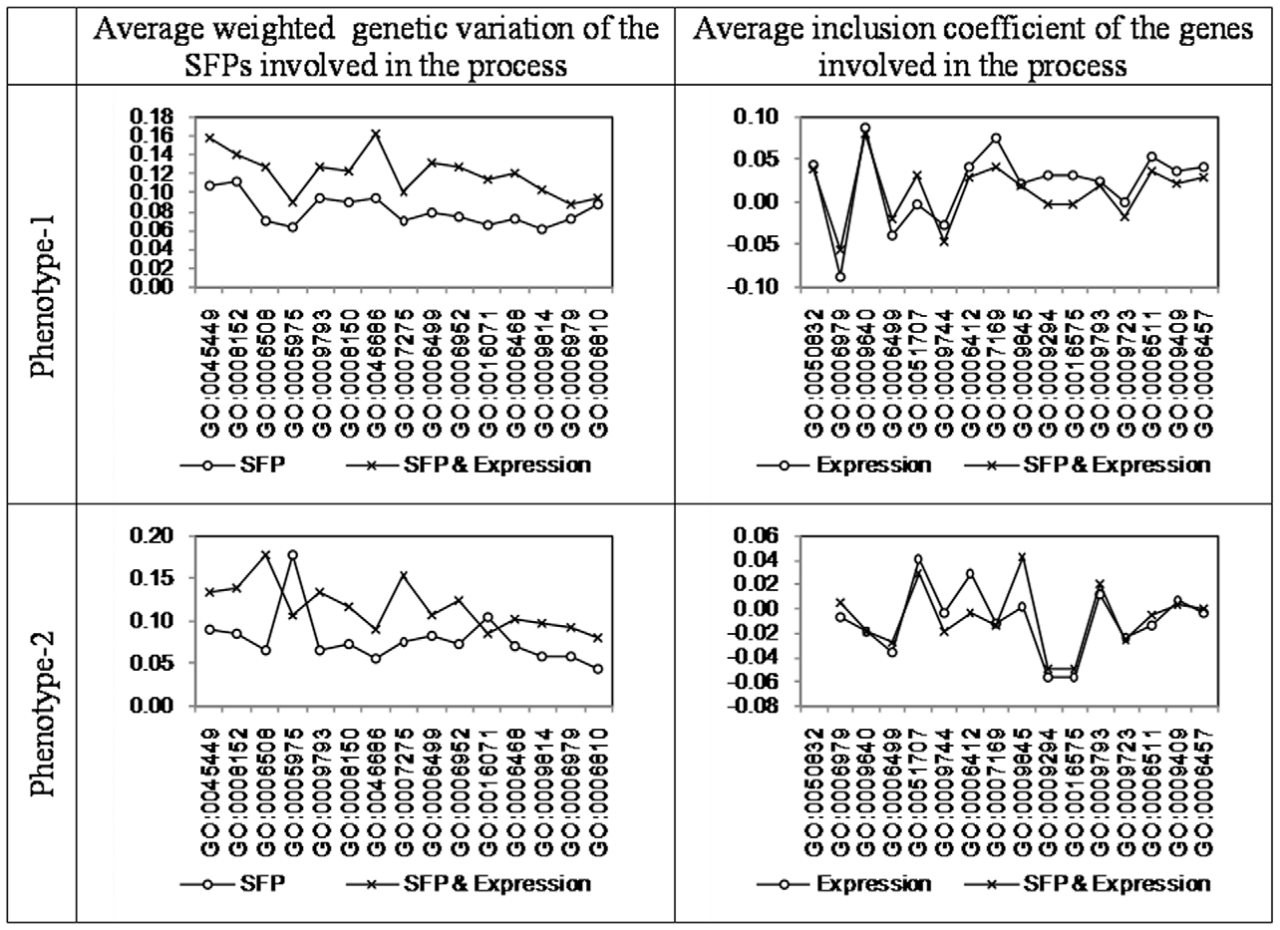
GO biological process enrichment estimated using indicator model (with 100 SFPs and/or 200 gene expressions).

For biological processes, the nature of the inclusion coefficients (corresponding to gene expressions) remained broadly the same in presence and in absence of SFPs in the model and this seems to the case for both phenotypes. (Average) weighted genetic variations of the SFPs however do differ with and without expression data in model. Also this departure is not similar for the two phenotypes (Figure 7). For phenotype-1 the effect is mostly in the magnitude where the pattern over different processes remaining the same. For phenotype-2 both the pattern and magnitude were affected.

**Figure 8:**
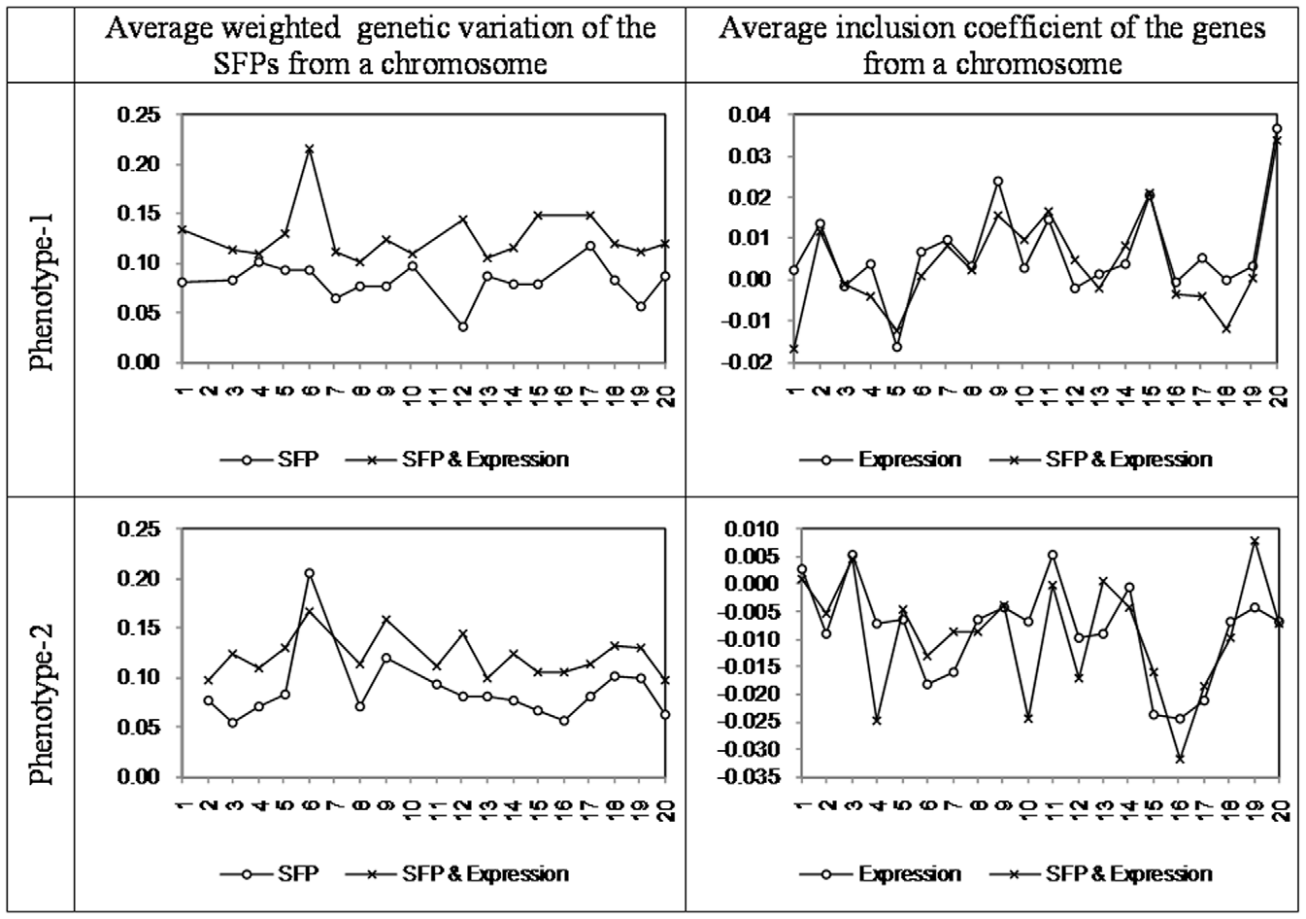
Chromosomal enrichment estimated using indicator model (with 100 SFPs and/or 200 gene expressions).

The comparative pattern of chromosomal level enrichment picture based on SFPs is somewhat opposite to what was observed for the biological processes. That is, the presence or absence of gene expression information in the predictive model affected to the magnitude of the weighted genetic variation for phenotype-2, whereas the estimates for phenotype-1 under the two models appeared to be roughly mirror images of each other (Figure 8).

As mentioned earlier the joint and marginal behaviors of the covariates need not be comparable. Also joint behavior depends on the presence of set of other covariates in the model. We have explored the joint and marginal nature of the different types of covariates with respect to their biological attributes. For example, gene ontological information on top SFPs and genes selected based on joint and marginal estimates of relevance were considered (Tables S1 and S3 in Supporting Information). For SFPs (treating each phenotype separately) three different relevance measures were considered 1) marginal *t*-test, 2) weighted genetic variation estimated from (indicator) model with SFPs only and 3) weighted genetic variation estimated from (indicator) model with SFPs and expression data. Similarly for gene expression data three different relevance measures were considered 1) correlation, 2) weighted inclusion coefficient estimated from (indicator) model with expression data only and 3) weighted inclusion coefficient estimated from (indicator) model with SFPs and expression data. SFPs in top ten according to any one of these measures were considered as top in the overall list. Also the biological processes annotated in the top covariates SFPs were also obtained (see Table S2 for SFPs and Table S4 for gene expression in Supporting Information).

## Discussion

Using RIL data of Soybean, we have compared different strategies to select important subset of SFPs for phenotype prediction using two different pathogen phenotypes. The ability to predict complex phenotypes from genotyping and/or gene expression is a keys aspect that could lead to personalized medicine. Our initial attempts to analyze this data and predict the phenotypes were found to be the best among those participating in DREAM5-Systems Biology B3 challenge.

Rapid advancements in laboratory techniques have made it possible to affordably produce large amount of genomic molecular marker and expression data. The simple statistical screening methods to find phenotype-genotype association or phenotype-expression association are still much used in practice because high dimensionality of the genomic data prevents use of more advanced statistical variable selection methods due to their computational demands.

The results indicate that indicator-based model without any preconditioning could provide perfect fit for the given data but might perform poorly when extrapolated for unseen data. However similar indicator models combined with judiciously carried out variable selection appeared to provide the best (or near best) predictive results for all data types considered in the illustration.

With increasing availability of genomic high-throughput data along with the shift in our objective of simple biomarker identification to phenotype prediction, the problems (in predictive ability of the model) as experienced here is expected to occur in other situations as well. That is we believe that these problems are not specific to the data or problem at hand. Thus we felt it would be useful to explore different intuitive and rigorous variable selection and/or data reduction techniques. Keeping in mind that there is also the added complexity of differing data sources/types it was essential to make these investigations context specific. Attempt was made to strike a balance in exploring different methods and use of different sources of information.

Certain peculiarities in the phenotype distributions were also noticed, in particular for the first phenotype. The first phenotype seemed to have heavier right tail. For the current analyses an *a-priori* Normal distribution was assumed which in combination of prior distributions of coefficients and other parameters in the model constituted a Student's *t*-like distribution with heavier tails. It is known that although *t*-type distributions allow more mass to the tails than the Normal distribution they are symmetric. However the first phenotype appears to be asymmetric when compared to the second phenotype measured from the same subjects. This could very well be the reason why predictive performance for the first phenotype is consistently poorer than the second phenotype in all the different data and variable selection efforts explored here. Thus, use of skewed distributions (like Gamma) might be worth exploring in the future.

Lack of stability among selected variables in high-dimensional data could influence the performance of any predictive model. Because a degree of variability (across samples) in data type like gene expression might be high, it is unrealistic to expect a finite (often small sized) learning set to truly capture/reflect the underlying variability. Thus more often than not test sets elements would fall outside the data domain (e.g. as is captured by the Regressor Variable Hull for multiple regression; [32]) of the learning set. While testing performance of a predictive model one might attempt to circumvent this issue by creating appropriate learning and test sets, where test set would not fall in the region of extrapolation compared to the sample in the learning set. For small set of selected regressors and with sufficiently large data this can be achieved by using the properties of Hat matrix [32]. However it is not suited for high-dimensional data as we have here and thus it is an open problem. A small demonstration has been provided in the Supporting Information (see Figure S2 and Text S1).

The methodological contribution of this paper is in exploration of variable selection techniques to alleviate the problem of over-fitting. All the models based in careful variable selection procedure were found to produce significant results based on permutation test. Different strategies based on the nature of covariates were explored and all methods were implemented under the Bayesian hierarchical modeling framework with indicator-based covariate selection.

## Supporting Information

Figure S1 Plots of gene-frequencies for different values of mean and variances of correlations. Bootstrap samples of size 200 (plants) were taken from the 260 plants. Correlation between each gene's expression and phenotype was calculated for each such subsample. Then for each gene mean and stander deviation (std.) over 100 such bootstrap subsamples were computed. Below top row pertains to phenotype-1 and bottom row pertains to phenotype-2. Note that the horizontal axis with negative values represents mean and the horizontal axis with positive values only represent the standard deviation. Left panel: Equidistant bin points were identified for mean and std., (%) frequency of genes in these categories were cross tabulated and plotted. Right panel: Equal percentile points were identified for mean and std., (%) frequency of genes in these categories were cross tabulated and plotted.
(TIF)

Figure S2 Correlations between out-of-sample predictions and corresponding observed values of phenotypes were computed for a wide range of models under two different learning set creation schemes. The two schemes for learning and testing creations are as follows. In the first scheme k( = 5) equal sized folds (of 52 samples) of the total sample (of 260) were created focusing on homogeneity of phenotypes only across the folds. In another scheme attempts were made to assure that the samples in test set are not in the region on of extrapolation of the learning set. This was done by partial homogenization of the samples using PCA. In this method the learning set was of size 208 and the test set was of 52. For data reduction and predictive model formation, Supervised principal component analysis (SPCA) followed by multiple regression was carried. Input variables in the regression model were selected based on 48 different cut-off values on correlation and 34 different choices on number of principal components to use as predictors. In all 1632 models were attempted for each scheme of test set creation. For both K-fold and split sample method, no information from test set was used to carry out data reduction or to form the predictive model. Top row-1 presents results for phenotype-1 and the bottom row those for phenotype-2. Note that the left pane presents results corresponding to 5 fold validation and the right panel presents those for split sample analysis.
(TIF)

Table S1 Gene Ontological information on top SFPs selected based on joint and marginal estimates of relevance. For each phenotype separately three different relevance measures were considered 1) marginal t-test, 2) weighted genetic variation estimated from (indicator) model with SFPs only and 3) weighted genetic variation estimated (indicator) model with SFPs and expression data. SFPs in top ten according to any one of these measures were considered as top in the overall list.
(DOC)

Table S2 The biological processes annotated in the top SFPs (see table S3 for description of top SFPs) with number of SFPs with the respective annotation.
(DOC)

Table S3 Gene Ontological information on top genes selected based on joint and marginal estimates of relevance. For each phenotype separately three different relevance measures were considered 1) correlation, 2) weighted inclusion coefficient estimated from (indicator) model with expression data only and 3) weighted inclusion coefficient estimated (indicator) model with SFPs and expression data. Genes in top twenty according to any one of these measures were considered as top in the overall list.
(DOC)

Table S4 The biological processes annotated in the top genes (see table S3 for description of top genes) with number of genes with the respective annotation.
(DOC)

Text S1 Contains further notes on supplementary Figures S1 and S2.
(DOC)
