# Supplementary figures and images for: A Bayesian Mixed Regression Based Prediction of Quantitative Traits from Molecular Marker and Gene Expression Data
Source: PLoS One. 2011 Nov 7;6(11):e26959. doi: 10.1371/journal.pone.0026959 (PMC3210128; doi:10.1371/journal.pone.0026959)

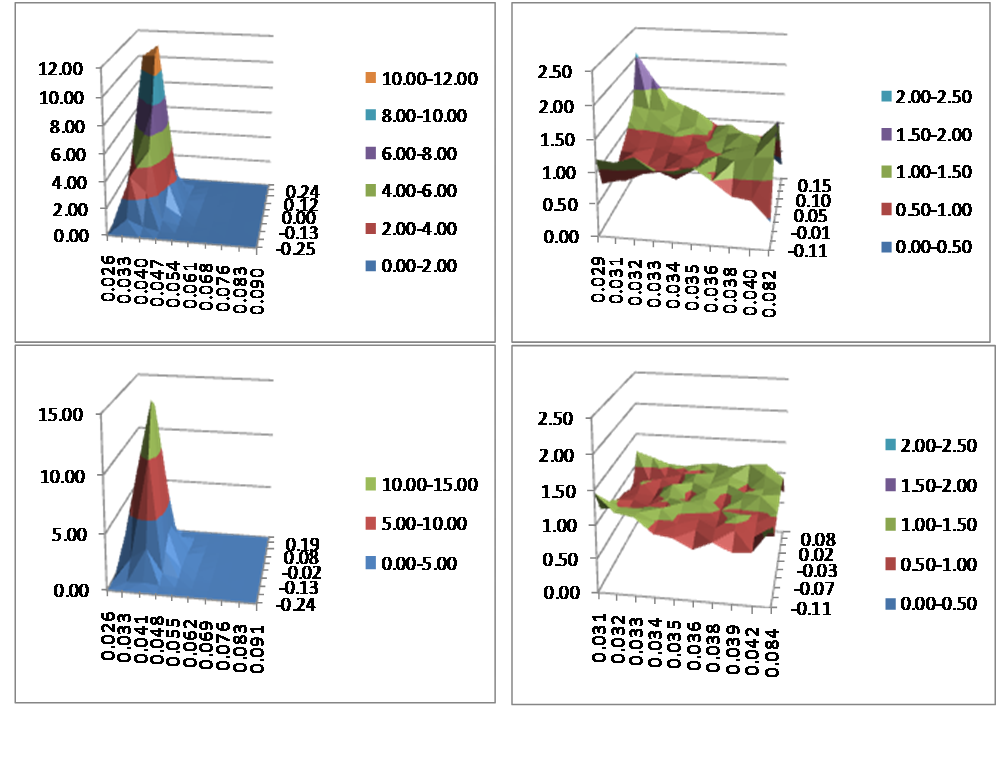

Supplement: Figure S1 — Plots of gene-frequencies for different values of mean and variances of correlations. Bootstrap samples of size 200 (plants) were taken from the 260 plants. Correlation between each gene's expression and phenotype was calculated for each such subsample. Then for each gene mean and stander deviation (std.) over 100 such bootstrap subsamples were computed. Below top row pertains to phenotype-1 and bottom row pertains to phenotype-2. Note that the horizontal axis with negative values represents mean and the horizontal axis with positive values only represent the standard deviation. Left panel: Equidistant bin points were identified for mean and std., (%) frequency of genes in these categories were cross tabulated and plotted. Right panel: Equal percentile points were identified for mean and std., (%) frequency of genes in these categories were cross tabulated and plotted. (TIF) [file pone.0026959.s001.tif]

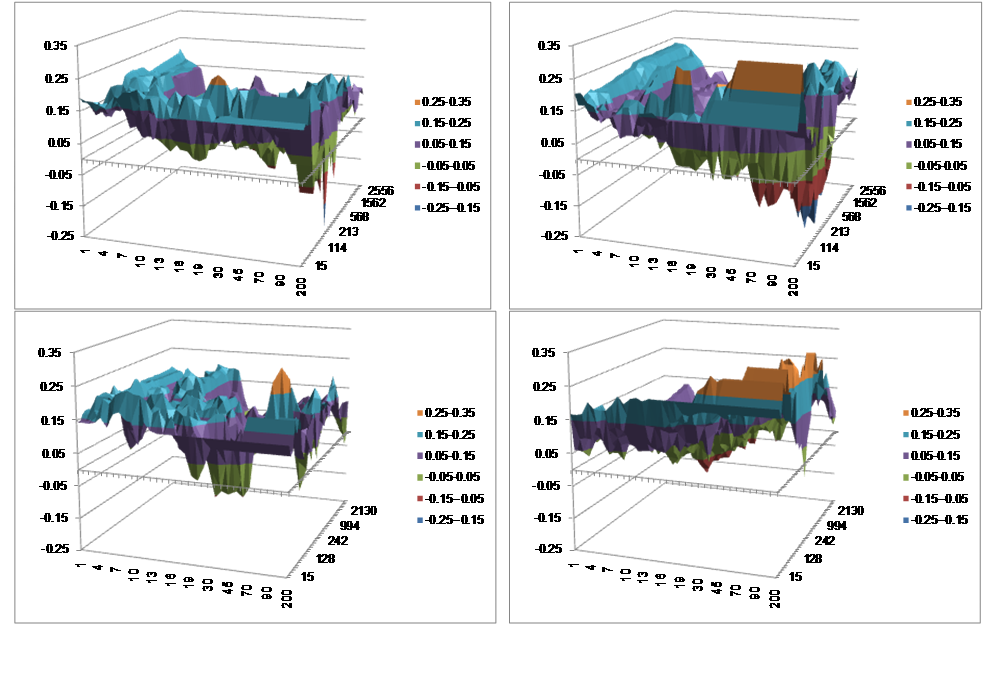

Supplement: Figure S2 — Correlations between out-of-sample predictions and corresponding observed values of phenotypes were computed for a wide range of models under two different learning set creation schemes. The two schemes for learning and testing creations are as follows. In the first scheme k( = 5) equal sized folds (of 52 samples) of the total sample (of 260) were created focusing on homogeneity of phenotypes only across the folds. In another scheme attempts were made to assure that the samples in test set are not in the region on of extrapolation of the learning set. This was done by partial homogenization of the samples using PCA. In this method the learning set was of size 208 and the test set was of 52. For data reduction and predictive model formation, Supervised principal component analysis (SPCA) followed by multiple regression was carried. Input variables in the regression model were selected based on 48 different cut-off values on correlation and 34 different choices on number of principal components to use as predictors. In all 1632 models were attempted for each scheme of test set creation. For both K-fold and split sample method, no information from test set was used to carry out data reduction or to form the predictive model. Top row-1 presents results for phenotype-1 and the bottom row those for phenotype-2. Note that the left pane presents results corresponding to 5 fold validation and the right panel presents those for split sample analysis. (TIF) [file pone.0026959.s002.tif]
