# Supplementary material for: A Bayesian Mixed Regression Based Prediction of Quantitative Traits from Molecular Marker and Gene Expression Data
Source: PLoS One. 2011 Nov 7;6(11):e26959. doi: 10.1371/journal.pone.0026959 (PMC3210128; doi:10.1371/journal.pone.0026959)
Supplement: Table S1 — Gene Ontological information on top SFPs selected based on joint and marginal estimates of relevance. For each phenotype separately three different relevance measures were considered 1) marginal t-test, 2) weighted genetic variation estimated from (indicator) model with SFPs only and 3) weighted genetic variation estimated (indicator) model with SFPs and expression data. SFPs in top ten according to any one of these measures were considered as top in the overall list. (DOC) [file pone.0026959.s003.doc]

| **Affymatrix ID** | **GO Process ID** | | **GO Process Term** | | | **Rank** | | | | |
| --- | --- | --- | --- | --- | --- | --- | --- | --- | --- | --- |
|  |  |  |  |  |  | T-test | | SFP model | | SFP & Expression model |
| **Phenotype-1** | | | | | | | | | | |
| Gma.7958.1.S1_at | GO:0008150 | | biological process | | | 1 | | 5 | | 35 |
| GmaAffx.79265.1.S1_at | GO:0006997 | | nucleus organization | | | 2 | | 4 | | 57 |
| Gma.4397.1.S1_at | GO:0010072 | | primary shoot apical meristem specification | | | 3 | | 2 | | 13 |
| Gma.12286.1.S1_at | GO:0006952 GO:0006499 | | defense response, N-terminal protein myristoylation | | | 4 | | 39 | | 15 |
| GmaAffx.2924.1.A1_at | NA | | NA | | | 5 | | 6 | | 1 |
| Gma.15940.1.A1_at | NA | | NA | | | 6 | | 68 | | 50 |
| Gma.2917.1.S1_at | GO:0015031 | | protein transport | | | 7 | | 76 | | 97 |
| GmaAffx.71174.1.S1_at | GO:0009061 GO:0006139 GO:0009117 | | anaerobic respiration, nucleobase, nucleoside, nucleotide and nucleic acid metabolic process, nucleotide metabolic process | | | 8 | | 1 | | 29 |
| Gma.5675.1.S1_at | GO:0008150 | | biological process | | | 9 | | 28 | | 24 |
| GmaAffx.42050.1.S1_at | NA | | NA | | | 10 | | 18 | | 25 |
| GmaAffx.68483.2.S1_at | NA | | NA | | | 13 | | 12 | | 6 |
| GmaAffx.90973.1.S1_at | GO:0046686 GO:0009793 | | response to cadmium ion, embryonic development ending in seed dormancy | | | 17 | | 7 | | 5 |
| Gma.5744.1.S1_at | NA | | NA | | | 19 | | 3 | | 3 |
| GmaAffx.51654.1.S1_at | GO:0006468 | | protein amino acid phosphorylation | | | 35 | | 98 | | 9 |
| GmaAffx.552.1.S1_at | GO:0009304 | | tRNA transcription | | | 36 | | 38 | | 10 |
| Gma.15151.2.A1_at | GO:0006950 | | response to stress | | | 41 | | 10 | | 55 |
| Gma.14666.1.S1_at | GO:0008152 | | metabolic process | | | 43 | | 8 | | 68 |
| Gma.8607.2.S1_at | GO:0007165 | | signal transduction | | | 73 | | 23 | | 8 |
| GmaAffx.15910.1.A1_at | GO:0009733 | | response to auxin stimulus | | | 76 | | 49 | | 4 |
| GmaAffx.37942.1.S1_at | NA | | NA | | | 80 | | 30 | | 7 |
| GmaAffx.36849.1.A1_at | GO:0008150 | | biological process | | | 81 | | 9 | | 18 |
| GmaAffx.792.3.S1_at | NA | | NA | | | 91 | | 26 | | 2 |
| **Phenotype-2** | | | | | | | | | | |
| GmaAffx.552.1.S1_at | | GO:0009304 | | tRNA transcription | 1 | | 1 | | 16 | |
| Gma.2831.1.S1_at | | GO:0045449 | | regulation of transcription | 2 | | 20 | | 4 | |
| GmaAffx.79265.1.S1_at | | GO:0006997 | | nucleus organization | 3 | | 6 | | 7 | |
| Gma.7791.1.S1_at | | GO:0045449 | | regulation of transcription, | 4 | | 44 | | 44 | |
| Gma.10557.1.A1_at | | GO:0006468 | | protein amino acid phosphorylation | 5 | | 37 | | 96 | |
| Gma.11344.1.S1_at | | GO:0006952 GO:0009626 GO:0009814 | | defense response, plant-type hypersensitive response, defense response, incompatible interaction | 6 | | 60 | | 46 | |
| GmaAffx.58109.1.S1_at | | NA | | NA | 7 | | 15 | | 3 | |
| Gma.773.1.S1_at | | GO:0008152 | | metabolic process | 8 | | 25 | | 61 | |
| GmaAffx.93183.1.S1_at | | GO:0006596 | | polyamine biosynthetic process | 9 | | 13 | | 29 | |
| Gma.3610.2.S1_at | | GO:0016071 | | mRNA metabolic process | 10 | | 21 | | 89 | |
| GmaAffx.87428.1.S1_at | | GO:0008150 | | biological process | 23 | | 8 | | 38 | |
| GmaAffx.49662.1.S1_at | | GO:0042545 | | cell wall modification | 24 | | 62 | | 2 | |
| GmaAffx.7201.1.S1_at | | GO:0008150 | | biological process | 30 | | 7 | | 21 | |
| Gma.2534.4.S1_at | | GO:0006508 | | proteolysis | 31 | | 65 | | 8 | |
| Gma.7800.1.A1_at | | NA | | NA | 33 | | 9 | | 1 | |
| GmaAffx.56562.1.S1_at | | NA | | NA | 34 | | 10 | | 62 | |
| GmaAffx.14229.1.S1_at | | GO:0008150 | | biological process | 37 | | 42 | | 10 | |
| Gma.2245.1.S1_at | | GO:0005975 | | carbohydrate metabolic process | 57 | | 4 | | 59 | |
| Gma.9957.1.S1_at | | GO:0008152 | | metabolic process | 63 | | 56 | | 5 | |
| GmaAffx.47108.1.S1_at | | GO:0006511 | | ubiquitin-dependent protein catabolic process | 71 | | 3 | | 98 | |
| GmaAffx.77599.1.S1_at | | GO:0006355 | | regulation of transcription, DNA-dependent | 78 | | 2 | | 11 | |
| Gma.16984.1.A1_at | | GO:0008150 | | biological process | 80 | | 63 | | 9 | |
| GmaAffx.71174.1.S1_at | | GO:0009061 GO:0006139 GO:0009117 | | anaerobic respiration, nucleobase, nucleoside, nucleotide and nucleic acid metabolic process, nucleotide metabolic process | 93 | | 5 | | 6 | |
