# Supplementary material for: A Bayesian Mixed Regression Based Prediction of Quantitative Traits from Molecular Marker and Gene Expression Data
Source: PLoS One. 2011 Nov 7;6(11):e26959. doi: 10.1371/journal.pone.0026959 (PMC3210128; doi:10.1371/journal.pone.0026959)
Supplement: Table S2 — The biological processes annotated in the top SFPs (see table S3 for description of top SFPs) with number of SFPs with the respective annotation. (DOC) [file pone.0026959.s004.doc]

| **GO term** | **GO_ID** | **Count for phenotype-1** | **Count for phenotype-2** | **Combined count** |
| --- | --- | --- | --- | --- |
| biological process | GO:0008150 | 3 | 4 | 7 |
| metabolic process | GO:0008152 | 1 | 2 | 3 |
| protein amino acid phosphorylation | GO:0006468 | 1 | 1 | 2 |
| defense response | GO:0006952 | 1 | 1 | 2 |
| regulation of transcription | GO:0045449 | 0 | 2 | 2 |
| nucleobase, nucleoside, nucleotide and nucleic acid metabolic process | GO:0006139 | 1 | 1 | 1 |
| nucleus organization | GO:0006997 | 1 | 1 | 1 |
| anaerobic respiration | GO:0009061 | 1 | 1 | 1 |
| nucleotide metabolic process | GO:0009117 | 1 | 1 | 1 |
| tRNA transcription | GO:0009304 | 1 | 1 | 1 |
| N-terminal protein myristoylation | GO:0006499 | 1 | 0 | 1 |
| embryonic development ending in seed dormancy | GO:0009793 | 1 | 0 | 1 |
| response to cadmium ion | GO:0046686 | 1 | 0 | 1 |
| response to stress | GO:0006950 | 1 | 0 | 1 |
| signal transduction | GO:0007165 | 1 | 0 | 1 |
| response to auxin stimulus | GO:0009733 | 1 | 0 | 1 |
| primary shoot apical meristem specification | GO:0010072 | 1 | 0 | 1 |
| protein transport | GO:0015031 | 1 | 0 | 1 |
| carbohydrate metabolic process | GO:0005975 | 0 | 1 | 1 |
| proteolysis | GO:0006508 | 0 | 1 | 1 |
| mRNA metabolic process | GO:0016071 | 0 | 1 | 1 |
| defense response, incompatible interaction | GO:0009814 | 0 | 1 | 1 |
| regulation of transcription, DNA-dependent | GO:0006355 | 0 | 1 | 1 |
| polyamine biosynthetic process | GO:0006596 | 0 | 1 | 1 |
| plant-type hypersensitive response | GO:0009626 | 0 | 1 | 1 |
| ubiquitin-dependent protein catabolic process | GO:0006511 | 0 | 1 | 1 |
| cell wall modification | GO:0042545 | 0 | 1 | 1 |
