# Supplementary material for: A Bayesian Mixed Regression Based Prediction of Quantitative Traits from Molecular Marker and Gene Expression Data
Source: PLoS One. 2011 Nov 7;6(11):e26959. doi: 10.1371/journal.pone.0026959 (PMC3210128; doi:10.1371/journal.pone.0026959)
Supplement: Table S3 — Gene Ontological information on top genes selected based on joint and marginal estimates of relevance. For each phenotype separately three different relevance measures were considered 1) correlation, 2) weighted inclusion coefficient estimated from (indicator) model with expression data only and 3) weighted inclusion coefficient estimated (indicator) model with SFPs and expression data. Genes in top twenty according to any one of these measures were considered as top in the overall list. (DOC) [file pone.0026959.s005.doc]

| **Affymatrix ID** | **GO Process ID** | **GO Process Term** | | **Rank** | | | |
| --- | --- | --- | --- | --- | --- | --- | --- |
|  |  |  |  | Correlation | | Expression model | SFP & Expression model |
| **Phenotype-1** | | | | | | | |
| Gma.17908.1.S1_s_at | GO:0006979 | response to oxidative stress | | 1 | | 199 | 193 |
| GmaAffx.82871.1.S1_at | GO:0008150 | biological process | | 2 | | 5 | 16 |
| GmaAffx.56989.1.S1_at | GO:0008150 | biological process | | 3 | | 11 | 20 |
| GmaAffx.65102.1.S1_at | GO:0008150 | biological process | | 4 | | 198 | 197 |
| Gma.7443.1.S1_a_at | GO:0006511 | ubiquitin-dependent protein catabolic process | | 5 | | 120 | 98 |
| Gma.15367.1.S1_at | GO:0008150 | biological process | | 6 | | 97 | 130 |
| Gma.6801.1.S1_at | GO:0008150 | biological process | | 7 | | 36 | 31 |
| GmaAffx.24349.1.A1_at | NA | NA | | 8 | | 12 | 9 |
| GmaAffx.86183.1.S1_at | NA | NA | | 9 | | 56 | 61 |
| GmaAffx.92801.1.S1_s_at | GO:0046686 GO:0008152 | response to cadmium ion, metabolic process | | 10 | | 21 | 14 |
| GmaAffx.67654.1.S1_at | NA | NA | | 11 | | 184 | 183 |
| GmaAffx.13732.1.A1_at | NA | NA | | 12 | | 148 | 167 |
| Gma.5243.1.S1_at | GO:0006468 | protein amino acid phosphorylation | | 13 | | 166 | 140 |
| GmaAffx.65469.1.S1_at | GO:0008150 | biological process | | 14 | | 149 | 134 |
| Gma.13141.1.S1_at | NA | NA | | 15 | | 76 | 60 |
| GmaAffx.83427.1.A1_at | GO:0008150 | biological process | | 16 | | 145 | 164 |
| Gma.3865.1.S1_at | GO:0008150 | biological process | | 17 | | 32 | 39 |
| GmaAffx.14960.1.S1_s_at | GO:0006355 | regulation of transcription, DNA-dependent | | 18 | | 109 | 143 |
| Gma.7443.1.S1_x_at | GO:0006511 | ubiquitin-dependent protein catabolic process | | 19 | | 1 | 3 |
| GmaAffx.36156.1.S1_at | GO:0008272 GO:0006810 | sulfate transport, transport | | 20 | | 71 | 160 |
| GmaAffx.614.1.S1_at | GO:0006412 | translation | | 32 | | 10 | 5 |
| GmaAffx.56379.1.S1_at | GO:0045449 | regulation of transcription | | 42 | | 22 | 7 |
| Gma.6867.1.S1_at | GO:0008150 | biological process | | 52 | | 48 | 6 |
| GmaAffx.40907.1.S1_at | GO:0008150 | biological process | | 71 | | 19 | 23 |
| Gma.17770.1.S1_at | GO:0007169 | transmembrane receptor protein tyrosine kinase signaling pathway | | 92 | | 3 | 15 |
| GmaAffx.79689.1.S1_at | GO:0007017 GO:0006457 GO:0007021 GO:0009793 GO:0000910 | microtubule-based process, protein folding, tubulin complex assembly, embryonic development ending in seed dormancy, cytokinesis | | 94 | | 27 | 17 |
| Gma.7484.1.S1_a_at | GO:0009416 GO:0009640 GO:0009651 | response to light stimulus, photomorphogenesis, response to salt stress | | 97 | | 2 | 2 |
| GmaAffx.92945.1.S1_x_at | GO:0009409 GO:0009414 GO:0009408 GO:0006970 GO:0009651 GO:0006355 GO:0042742 GO:0010120 GO:0010120 GO:0050832 | response to cold, response to water deprivation, response to heat, response to osmotic stress, response to salt stress, regulation of transcription, DNA-dependent, defense response to bacterium, camalexin biosynthetic process, camalexin biosynthetic process, defense response to fungus | | 106 | | 8 | 13 |
| GmaAffx.18372.1.S1_at | GO:0006535 | cysteine biosynthetic process from serine | | 109 | | 13 | 8 |
| Gma.16613.1.S1_at | GO:0009409 GO:0042538 | response to cold, hyperosmotic salinity response | | 115 | | 20 | 19 |
| Gma.7237.2.A1_at | NA | NA | | 119 | | 4 | 1 |
| Gma.5192.1.S1_at | GO:0032313 | regulation of Rab GTPase activity | | 131 | | 6 | 11 |
| GmaAffx.30428.1.S1_at | GO:0009408 GO:0006457 | response to heat, protein folding | | 138 | | 9 | 45 |
| Gma.409.1.A1_at | GO:0008150 | biological process | | 140 | | 17 | 4 |
| GmaAffx.18779.1.S1_at | NA | NA | | 146 | | 18 | 77 |
| Gma.2382.1.S1_at | GO:0009414 GO:0009695 GO:0009611 GO:0009753 GO:0051707 | response to water deprivation, jasmonic acid biosynthetic process, response to wounding, response to jasmonic acid stimulus, response to other organism | | 154 | | 45 | 18 |
| GmaAffx.54236.1.S1_at | GO:0051707 | response to other organism, | | 164 | | 146 | 10 |
| Gma.12452.1.A1_at | GO:0008150 | biological process, | | 165 | | 16 | 36 |
| GmaAffx.92945.1.S1_at | GO:0009409 GO:0009414 GO:0009408 GO:0006970 GO:0009651 GO:0006355 GO:0042742 GO:0010120 GO:0010120 GO:0050832 | response to cold, response to water deprivation, response to heat, response to osmotic stress, response to salt stress, regulation of transcription, DNA-dependent, defense response to bacterium, camalexin biosynthetic process, camalexin biosynthetic process, defense response to fungus | | 184 | | 7 | 21 |
| Gma.7346.1.S1_at | GO:0006468 | protein amino acid phosphorylation | | 198 | | 14 | 67 |
| Gma.17559.1.S1_at | GO:0030001 | metal ion transport | | 199 | | 15 | 12 |
| **Phenotype-2** | | | | | | | |
| Gma.17169.1.A1_at | GO:0009411 GO:0006290 | response to UV, pyrimidine dimer repair | 1 | | 195 | | 180 |
| Gma.5225.1.A1_at | GO:0006499 | N-terminal protein myristoylation | 2 | | 200 | | 192 |
| Gma.2581.1.S1_a_at | GO:0006499 GO:0006468 | N-terminal protein myristoylation, protein amino acid phosphorylation | 3 | | 159 | | 179 |
| GmaAffx.71265.1.S1_at | GO:0009294 GO:0016575 | DNA mediated transformation, histone deacetylation | 4 | | 198 | | 198 |
| GmaAffx.73856.1.S1_at | GO:0006350 | transcription | 5 | | 42 | | 143 |
| GmaAffx.6888.1.S1_at | GO:0008150 | biological process | 6 | | 169 | | 148 |
| Gma.18071.1.S1_at | GO:0008150 | biological process | 7 | | 138 | | 116 |
| Gma.12045.1.S1_at | GO:0009063 GO:0009416 GO:0009646 GO:0009744 GO:0043617 | amino acid catabolic process, response to light stimulus, response to absence of light, response to sucrose stimulus, cellular response to sucrose starvation | 8 | | 41 | | 158 |
| Gma.7484.1.S1_a_at | GO:0009416 GO:0009640 GO:0009651 | response to light stimulus, photomorphogenesis, response to salt stress | 9 | | 165 | | 155 |
| Gma.9412.1.S1_at | GO:0046686 GO:0009657 GO:0006461 | response to cadmium ion, plastid organization, protein complex assembly | 10 | | 5 | | 37 |
| Gma.6079.1.S1_at | GO:0008150 | biological process | 11 | | 142 | | 183 |
| GmaAffx.1856.1.A1_at | NA | NA | 12 | | 168 | | 99 |
| GmaAffx.49784.1.A1_at | GO:0006355 GO:0009737 GO:0009739 GO:0009753 GO:0009751 GO:0009785 | regulation of transcription, DNA-dependent, response to abscisic acid stimulus, response to gibberellin stimulus, response to jasmonic acid stimulus, response to salicylic acid stimulus, blue light signaling pathway | 13 | | 106 | | 46 |
| GmaAffx.82653.1.S1_at | NA | NA | 14 | | 15 | | 35 |
| Gma.7683.1.S1_at | GO:0009733 GO:0010051 GO:0010228 | response to auxin stimulus, xylem and phloem pattern formation, vegetative to reproductive phase transition | 15 | | 146 | | 88 |
| Gma.12080.1.S1_at | GO:0016117 | carotenoid biosynthetic process | 16 | | 64 | | 79 |
| GmaAffx.69199.2.S1_at | NA | NA | 17 | | 96 | | 132 |
| GmaAffx.31738.1.A1_at | GO:0009627 GO:0009410 | systemic acquired resistance, response to xenobiotic stimulus | 18 | | 102 | | 125 |
| GmaAffx.65469.1.S1_at | GO:0008150 | biological process | 19 | | 11 | | 15 |
| Gma.13231.1.A1_at | GO:0046907 | intracellular transport | 20 | | 179 | | 185 |
| GmaAffx.33198.1.S1_at | GO:0008150 | biological process | 28 | | 9 | | 23 |
| GmaAffx.57607.1.S1_at | GO:0006807 | nitrogen compound metabolic process | 34 | | 16 | | 45 |
| GmaAffx.62679.1.S1_at | GO:0006505 | GPI anchor metabolic process | 38 | | 4 | | 1 |
| Gma.1328.1.S1_at | GO:0000025 GO:0000023 GO:0005976 GO:0005983 | maltose catabolic process, maltose metabolic process, polysaccharide metabolic process, starch catabolic process | 40 | | 133 | | 9 |
| GmaAffx.73629.1.S1_at | NA | NA | 49 | | 31 | | 14 |
| GmaAffx.85125.1.S1_at | GO:0008150 | biological process | 52 | | 118 | | 16 |
| Gma.2590.4.S1_a_at | GO:0019252 GO:0048573 | starch biosynthetic process, photoperiodism, flowering | 68 | | 10 | | 4 |
| Gma.4885.1.S1_at | GO:0008150 | biological process | 78 | | 6 | | 3 |
| Gma.13135.1.S1_at | GO:0042335 | cuticle development | 81 | | 2 | | 29 |
| Gma.15483.2.S1_a_at | GO:0046686 GO:0005975 | response to cadmium ion, carbohydrate metabolic process | 104 | | 23 | | 10 |
| Gma.9531.1.S1_at | GO:0046686 | response to cadmium ion | 111 | | 73 | | 18 |
| Gma.13002.1.S1_at | NA | NA | 112 | | 13 | | 26 |
| Gma.4408.2.S1_at | GO:0006633 GO:0006108 GO:0009845 GO:0009793 | fatty acid biosynthetic process, malate metabolic process, seed germination, embryonic development ending in seed dormancy | 113 | | 21 | | 2 |
| Gma.4368.1.S1_at | NA | NA | 121 | | 36 | | 17 |
| Gma.913.1.A1_at | GO:0009409 | response to cold | 125 | | 14 | | 22 |
| GmaAffx.9345.1.S1_at | GO:0001522 | pseudouridine synthesis | 135 | | 12 | | 5 |
| Gma.4445.1.S1_at | GO:0008150 | biological process | 155 | | 81 | | 12 |
| GmaAffx.62051.1.S1_at | GO:0000105 | histidine biosynthetic process | 157 | | 1 | | 8 |
| Gma.6247.1.A1_at | GO:0006355 | regulation of transcription, DNA-dependent | 158 | | 19 | | 7 |
| GmaAffx.614.1.S1_at | GO:0006412 | translation | 159 | | 7 | | 93 |
| Gma.7937.1.S1_at | GO:0008150 | biological process | 166 | | 20 | | 87 |
| Gma.16291.1.S1_at | GO:0008150 | biological process | 167 | | 3 | | 19 |
| GmaAffx.28578.2.S1_at | GO:0051707 | response to other organism | 173 | | 8 | | 13 |
| Gma.3405.2.S1_s_at | GO:0009081 GO:0008152 | branched chain family amino acid metabolic process, metabolic process | 181 | | 17 | | 11 |
| Gma.2387.2.A1_at | GO:0006555 | methionine metabolic process | 188 | | 46 | | 20 |
| GmaAffx.92718.1.S1_s_at | GO:0006413 | translational initiation | 197 | | 18 | | 6 |
