# Supplementary material for: A Bayesian Mixed Regression Based Prediction of Quantitative Traits from Molecular Marker and Gene Expression Data
Source: PLoS One. 2011 Nov 7;6(11):e26959. doi: 10.1371/journal.pone.0026959 (PMC3210128; doi:10.1371/journal.pone.0026959)
Supplement: Text S1 — Contains further notes on supplementary Figures S1 and S2. (DOC) [file pone.0026959.s007.doc]

**A Bayesian Mixed Regression based Prediction of Quantitative Traits from Molecular Marker and Gene Expression Data**

Madhuchhanda Bhattacharjee^1*^ and Mikko J. Sillanpää^2-5^

^1^ Department of Statistics, University of Pune, Pune, Maharashtra, India

^2^ Department of Mathematics and Statistics, University of Helsinki, Helsinki, Finland

^3^ Department of Agricultural Sciences, University of Helsinki, Helsinki, Finland

^4^ Department of Mathematical Sciences, University of Oulu, Oulu, Finland

^5^ Department of Biology, University of Oulu, Oulu, Finland

**Supplementary Information**

**Note for figure S1**: From left panel we observe that contours are near elliptical. This indicates possible independence between mean and standard deviation of correlations. This is further confirmed by the figures in right panel. Right panel figures show if (1) genes with top correlation are selected then there will be all varied range of variability (over samples) in them,

(2) If gene with low std (i.e. stable) genes are to be chosen then high correlation with phenotype can’t be guaranteed particularly for phenotype-2.

**Note for figure S2**: For both phenotypes out-of-data prediction quality has improved over KF results to the split sample based results. Below are the correlations based on predictions under different validation schemes:

K-fold: Phenotype-1: 0.279 Phenotype-2: 0.323

Split-sample: PCA based Phenotype-1: 0.331 Phenotype-2: 0.344

Split-sample: Correlation based Phenotype-1: 0.354 Phenotype-2: 0.386
